# Exploring the visual world of fossilized and modern fungus gnat eyes (Diptera: Keroplatidae) with X-ray microtomography

**DOI:** 10.1098/rsif.2019.0750

**Published:** 2020-02-05

**Authors:** Gavin J. Taylor, Stephen A. Hall, Johan A. Gren, Emily Baird

**Affiliations:** 1 Department of Biology, Lund University, Lund, Sweden; 2 Division of Solid Mechanics, Lund University, Lund, Sweden; 3 Department of Geology, Lund University, Lund, Sweden; 4 Department of Zoology, Stockholm University, Stockholm, Sweden

**Keywords:** eye evolution, visual specialization, optical analysis, X-ray microtomography, insect, fossil

## Abstract

Animal eyes typically possess specialized regions for guiding different behavioural tasks within their specific visual habitat. These specializations, and evolutionary changes to them, can be crucial for understanding an animal's ecology. Here, we explore how the visual systems of some of the smallest flying insects, fungus gnats, have adapted to different types of forest habitat over time (approx. 30 Myr to today). Unravelling how behavioural, environmental and phylogenetic factors influence the evolution of visual specializations is difficult, however, because standard quantitative techniques often require fresh tissue and/or provide data in eye-centric coordinates that prevent reliable comparisons between species with different eye morphologies. Here, we quantify the visual world of three gnats from different time periods and habitats using X-ray microtomography to create high-resolution three-dimensional models of the compound eyes of specimens in different preservation states—fossilized in amber, dried or stored in ethanol. We present a method for analysing the geometric details of individual corneal facets and for estimating and comparing the sensitivity, spatial resolution and field of view of species across geographical space and evolutionary time. Our results indicate that, despite their miniature size, fungus gnats do have variations in visual properties across their eyes. We also find some indication that these visual specializations vary across species and may represent adaptations to their different forest habitats. Overall, the findings demonstrate how such investigations can be used to study the evolution of visual specializations—and sensory ecology in general—across a range of insect taxa from different geographical locations and across time.

## 1. Introduction

Vision provides essential information for guiding the behaviour of many animals and is often critical for orchestrating both movement through and interactions with their environment [1]. To meet the sensory demands of visually guided behaviour, animal eyes typically contain specialized regions of high resolution and/or sensitivity that are optimized for acquiring information to guide specific behavioural tasks, such as pursuing conspecifics or detecting flowers. Another, less well understood, factor affecting eye adaptations is the visual environment itself, which may have features that vary dramatically between habitats—consider, for example, the difference in visual information available in a bright open meadow compared with that of a dim cluttered rainforest [2]. To acquire the visual information necessary for guiding behaviour in different environments, animal eyes are likely to have habitat-specific specializations. For example, Scales & Butler [3] found that differences in visual specializations of several damselfly species could indeed be linked to the visual properties of each species' preferred microhabitat. To date, knowledge about the relationship between vision and habitat and the evolutionary factors shaping visual specializations is poor, yet it is critical if we are to understand the sensory ecology of different species and to predict if/how they will adapt to and survive both long- and short-term habitat changes.

Fungus gnats are interesting models for studying the evolution of visual specializations because they have eyes that comprise just a few hundred lenses and brains the size of a grain of salt, but they are nonetheless capable of using vision to guide behaviour in forests, which are arguably some of the most visually complex environments on the Earth [4]. How are the diminutive eyes of these insects capable of providing sufficient information to guide behaviour in their challenging visual habitat? Species of fungus gnats inhabit a broad range of forest types, from the Arctic to the tropics, and their frequent occurrence in amber shows the tribe has thrived for at least 50 Myr [5]. Their long evolutionary success and their ability to occupy a range of complex visual habitat types must, at least in part, be attributed to each species’ capacity to acquire the visual information necessary to control behaviour. Indeed, the eye specializations evolved for one habitat may not to be optimal for other visual environments because of differences, for example, in light intensity and openness. Do different species have visual specializations that reflect differences in their preferred forest habitat? In terms of total illumination, tropical rainforest floors can be a factor of 4 dimmer than in temperate rainforests (with approximately 0.5% and 2% of the incident light at the canopy reaching the ground, respectively) [6]. Finer details of the visual landscape are also likely to vary between forest types; above 70° elevation, dense overstories provide more canopy cover than sparse overstories (up to 30% more at the zenith) [7]. Furthermore, although scene complexity has not been directly compared between forest types, tropical forests are also likely to be more visually cluttered because the lower storey can have roughly 50–200% more leaf area than temperate, deciduous forests (cumulative leaf area index < 10 m; tropical: 2.5, temperate: 0.75 and 1.5) [8–10]. Considering this, we hypothesize that the visual specializations of the fungus gnats from tropical habitats would be more similar to each other than to a species from a temperate forest habitat, despite the distance between their geographical and temporal distributions. Specifically, given that tropical forests are relatively dark as a result of their dense canopies, we expect tropical gnats to have visual specializations that improve their optical sensitivity. We predict that these increases in optical sensitivity will be particularly prominent in the dorsal visual field to compensate for the additional canopy cover in dense forests.

To begin to test these hypotheses, we need to compare the visual specializations of extant species from different locations/habitats and extinct species from different points in environmental history from those same locations. Such comparisons are also necessary if we are to understand the evolutionary history of not only vision but sensory systems in general, and to make reliable predictions about how different species will respond to future habitat change [3]. Because the structural properties of insect corneas—which can be used to identify visual specializations—are often preserved naturally on samples in taxonomic collections [11,12], these collections are an ideal resource to study factors driving the evolution of visual specializations of fungus gnats (and other species). Unfortunately, current methods for analysing the visual properties of corneas are unsuitable for such investigations because (i) some methods require fresh tissue and are destructive, so they can only be applied to readily available and extant species [13], and (ii) most methods only provide a two-dimensional representation of the compound eye and thus limit our ability to understand how it viewed its three-dimensional world [14,15]. To overcome some of these limitations, we recently developed a method to quantify and recreate the visual world of arthropod eyes based on data from X-ray microtomography (microCT)—a popular tool for morphological and structural analysis in many areas of biology [16]. A remaining limitation of this method, however, is that it relies on analyses of volumetric data obtained from specially prepared fresh specimens (i.e. from extant species) [17].

To analyse the visual world of fungus gnat species from different geographical locations and evolutionary time periods, we further developed our method such that, rather than requiring volumetric data from specially prepared fresh samples, we are now able to analyse the corneal surface of specimens from taxonomic collections in different states of preservation, from naturally preserved specimens that are fossilized in amber or dried and from specimens preserved in ethanol. With our method, visual specializations are analysed using the shape of the cornea and the geometry of the individual facets within it. In particular, we calculate the local inter-facet (IF) angle and facet diameter (indications of visual resolution and sensitivity, respectively), as well as the extent of its corneal projection (CP) as an approximation of the field of view [17]. These data can then be represented with respect to not only the local coordinates on the eye but also world-based coordinates. This not only enables us to better understand how the animals perceived their visual world, but also facilitates direct comparisons between species or even taxa with eyes of different morphologies or sizes and in different preservation states. Because our method is non-destructive and is based only on the corneal structure, it is suitable to perform on fragile samples such as three-dimensionally preserved fossils or museum specimens, enabling the use of historical material for investigations into the factors that drive and shape the visual evolution of compound eyes. Note that, while taxonomists have used microCT scans to create three-dimensional models of the heads from historical insect samples for nearly two decades (e.g. [18]), our study represents a novel use of this technology by using such models to calculate visual parameters of compound eyes and to provide data on their vision. As far as we know, this has only previously been available for extant insects analysed using the pseudopupil [19,20] or corneal reflection [21] methods.

To begin to explore whether the miniature eyes of fungus gnat species have evolved habitat-specific visual specializations, we apply our methodology for analysing the corneal structure of compound eyes to specimens from different time periods and geographical locations. We focus our study on females from three species of the cosmopolitan Orfeliini tribe from different habitats and evolutionary time periods: (i) an ancient species from the Orfeliini tribe (electronic supplementary material, figure S1) from the tropical rainforests of Scandinavia approximately 30 Ma, (ii) an extant species from the tropical rainforests of Africa (*Rutylapa* sp.), and (iii) an extant species from the temperate forests of modern Scandinavia (*Neoplatyura modesta*). While both Scandinavian gnats are from similar geographical locations, they represent species from different habitat types, as forests in the Baltic region have thinned substantially since the Eocene [22]. Using these specimens, we can begin to compare the visual specializations of extant species from different locations/habitats and extinct species from different points in environmental history from those same locations. Such comparisons are necessary if we are to understand the evolution of not only vision but sensory systems in general and to make reliable predictions about how different species will respond to future habitat change [3].

## 2. Methods and materials

### 2.1. Animals

Each of the three specimens of fungus gnat species used in the study (*N. modesta*, *Rutylapa* sp. and an orfeliine species) belong to the Orfeliini tribe and were preserved in different ways—dried, in ethanol or as an amber endocast (table 1). As the males of many species of the order Diptera have evolved visual specializations related to pursuit flights during courtship [23], we selected only females for our analysis to minimize the effect of sexual dimorphism. The Entomology Collection at the Department of Biology, Lund University, Lund, Sweden, provided dried and pinned samples of *N. modesta*, the Natural History Museum at the University of Oslo, Oslo, Norway, provided samples of *Rutylapa* sp. preserved in ethanol, and the Department of Geology at Lund University provided a piece of amber containing inclusions of a species from the Orfeliini tribe (electronic supplementary material, figure S1). Although shrinkage can occur in the tissue of dried and ethanol-preserved insect specimens [24], this is least apparent in the morphology of the exoskeleton, and a previous comparison between sample preservation methods indicated that ethanol fixation and drying do not generate obvious changes in the three-dimensional appearance of dipteran corneas [25] (GJ Taylor 2018, personal observation). Before imaging, we examined samples from the collections using a light microscope, firstly, to select similarly sized individuals (the samples varied in head width by approximately 20%) (table 1) and, secondly, to check that obvious distortions in the cuticle were not present.

**Table 1.** Summary of parameters related to each fungus gnat specimen.

species	*Neoplatyura modesta*	orfeliine species	*Rutylapa* sp.
habitat	temperate forest	tropical forest (Eocene)	tropical forest
collection location and year	Stenshuvud, Sweden (1977)	Lilla Beddinge, Sweden (1990)	West Usambara Mountains, Tanzania (1990)
preservation method	dried	endocast in amber	70% ethanol
head width (μm)	452	519	547

### 2.2. X-ray microtomography

MicroCT was performed on the head of each sample using a Zeiss XRM520 at the 4D Imaging Lab at Lund University. For each dataset, X-ray projections were obtained over 360° (see electronic supplementary material, table S1 for scanning parameters and electronic supplementary material, table S2 for DOIs of datasets) and reconstructed into three-dimensional volumes with 1 µm isotropic resolution. Because of their different preservation states, each sample was mounted in the tomograph using a different method, as outlined below.

The pin of the dried sample was clamped in a pin-vice; the pin and sample were then mounted directly in the tomograph and oriented such the pin did not occlude the projection of the gnat head on the detector.

The ethanol-preserved sample was placed at the bottom of a 0.5 ml microcentrifuge tube (Eppendorf) and partially covered with a small amount of 70% ethanol (the surface tension of the liquid held the gnat in place). A ball of Parafilm was then used to fill the remaining volume of the tube to prevent evaporation of the ethanol. The initial tube was placed partially within a larger 2 ml microcentrifuge tube and secured with Parafilm, and the latter was clamped in a pin-vice and mounted in the tomograph.

The amber block was hot-glued to a pin that was clamped in a pin-vice and mounted in the tomograph. A lower resolution scan was initially conducted on the block to identify the location of the inclusion; the head was then scanned at 1 µm resolution.

### 2.3. General analysis procedure

**Figure 1.**
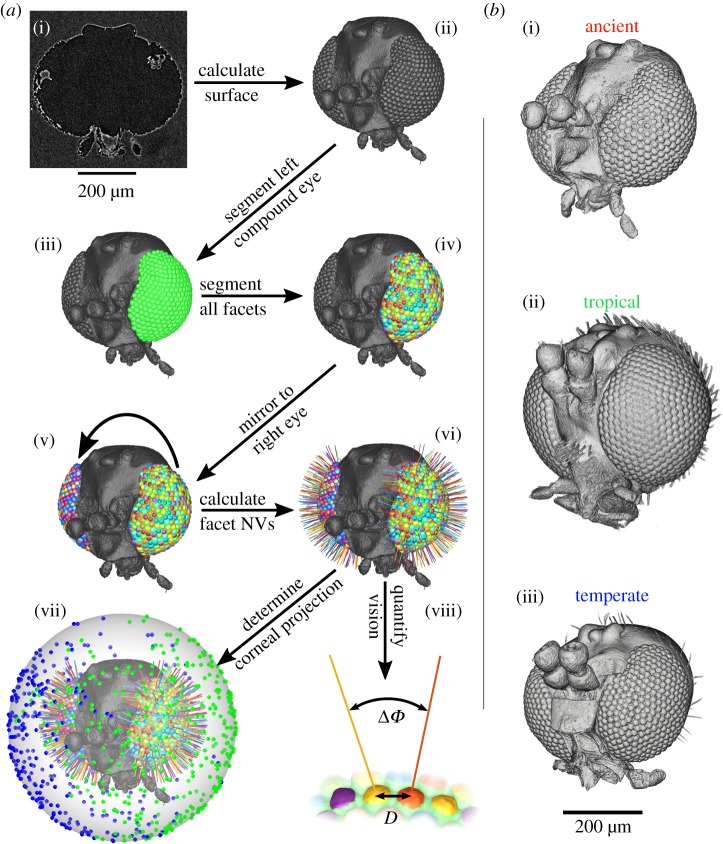
(*a*) Workflow to quantify the visual parameters of an insect cornea from X-ray microtomography (see Baird & Taylor [16] for a general overview of the imaging process). (i) Virtual section through a reconstructed volume showing the head of an Eocene fungus gnat from an amber endocast. (ii) The reconstructed exterior surface of the gnat's head. (iii) The segmented left compound eye, and (iv) all of its segmented corneal facets. (v) The left eye mirrored to the right-hand side of the head, and (vi) the calculated normal vector (NV) from all facet surfaces. (vii) The NVs are projected onto a sphere to determine their viewing directions and visual fields (green, left eye; blue, right eye). (viii) Visual parameters can be calculated across the eye: facet diameter (*D*) and IF angle (Δ*Φ*). (*b*) Volume renderings of each gnat imaged for this study. (i) Endocast of a species from the Orfeliini tribe in Eocene Baltic amber (note: the indentation on the anterodorsal region of the compound eye does not appear to be a preservation artefact as it occurs symmetrically on both eyes, which are separated by a raised ridge along the frons; see also electronic supplementary material, figure S1); (ii) ethanol-preserved head of *Rutylapa* sp.; and (iii) dried head of *Neoplatyura modesta*. The scale bar underneath (*b*(iii)) applies to all heads in (*b*). (Online version in colour.)

The volume rendering from microCT scans allowed the facets of the compound eye to be individually identified (figure 1*a*(i)) and a digital representation of the entire head surface to be computed (figure 1*a*(ii)) in Amira (Thermo Fischer Scientific, USA). The border of the left eye (figure 1*a*(iii)) and each of its facets (figure 1*a*(iv)) were then manually labelled by selecting paths across the surface. This isolated the surface of every individual facet on the left eye and the right eye was represented by mirroring the surface of the left eye (figure 1*a*(v)). For additional information on these steps, see the ‘Detailed surface analysis procedure’ in the electronic supplementary material.

**Figure 2.**
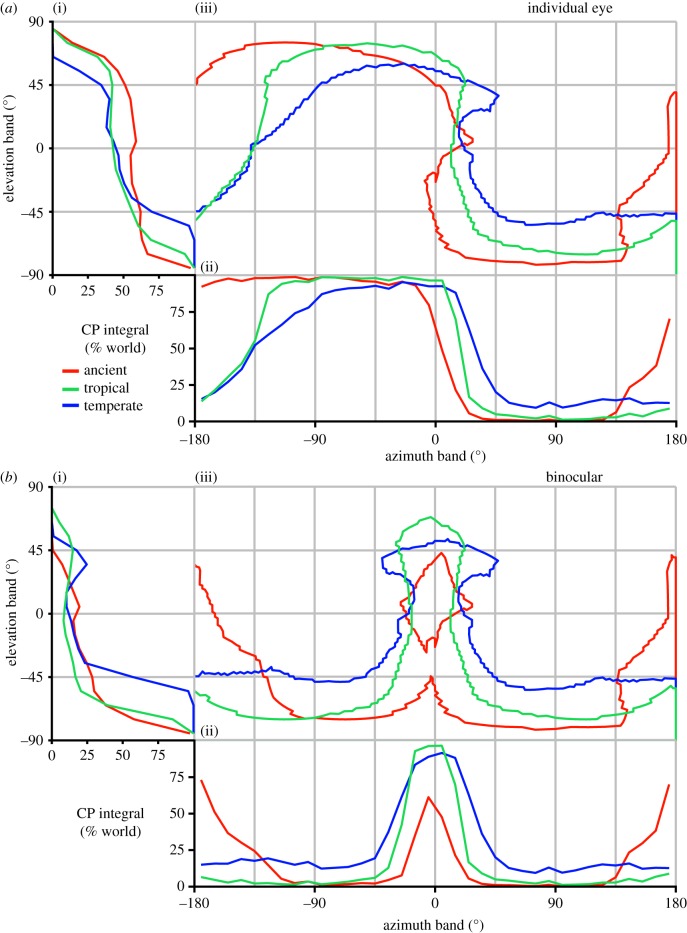
Comparison of the extent of the corneal projection (CP) between fungus gnat species. The monocular CP of each gnat's left eye projected onto the visual world using an equirectangular projection as if each gnat is facing towards 0° azimuth (*a*(iii)). The CP was integrated across azimuth points in the eye's CP to provide an elevation profile for average IF angle (*a*(i)) or, conversely, integrated over elevation to provide an azimuth profile (*a*(ii)). The binocular CP is depicted in (*b*). Red, green and blue lines represent the ancient fungus gnat, the extant tropical fungus gnat and the extant temperate fungus gnat species, respectively. (Online version in colour.)

**Figure 3.**
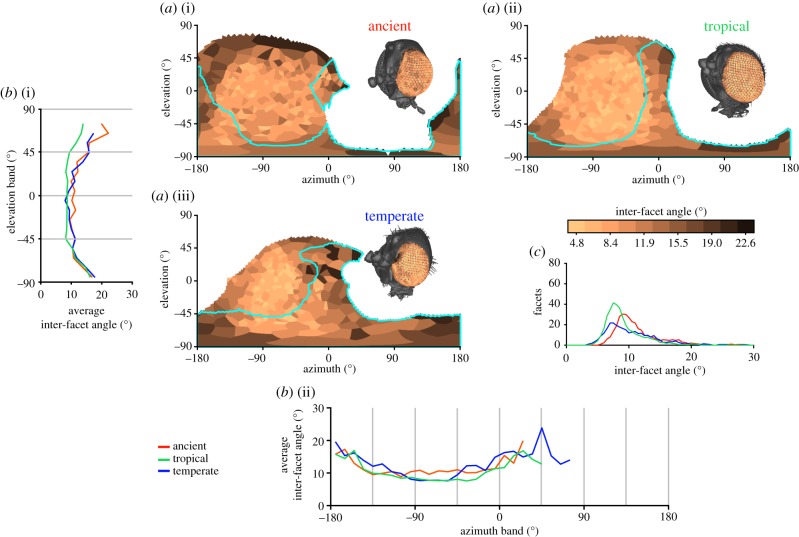
Comparison of the IF angle between fungus gnat species. The distribution of IF angles for the ancient fungus gnat (*a*(i)), the extant tropical fungus gnat (*a*(ii)) and the extant temperate fungus gnat (*a*(iii)). IF angles are projected onto the visual world using an equirectangular projection as if each gnat is facing towards 0° azimuth. The colour map of IF angle is also mapped onto the corneal facets in the inset of each panel, and the cyan lines indicate the limit of binocular corneal projection (CP). The IF angle was averaged across azimuth points in the eye's CP to provide an elevation profile for average IF angle (*b*(i)) or, conversely, averaged over elevation to provide an azimuth profile (*b*(ii)). The frequency distribution of IF angles for each sample is shown in (*c*). Lower IF angles indicate higher resolution vision. (Online version in colour.)

**Figure 4.**
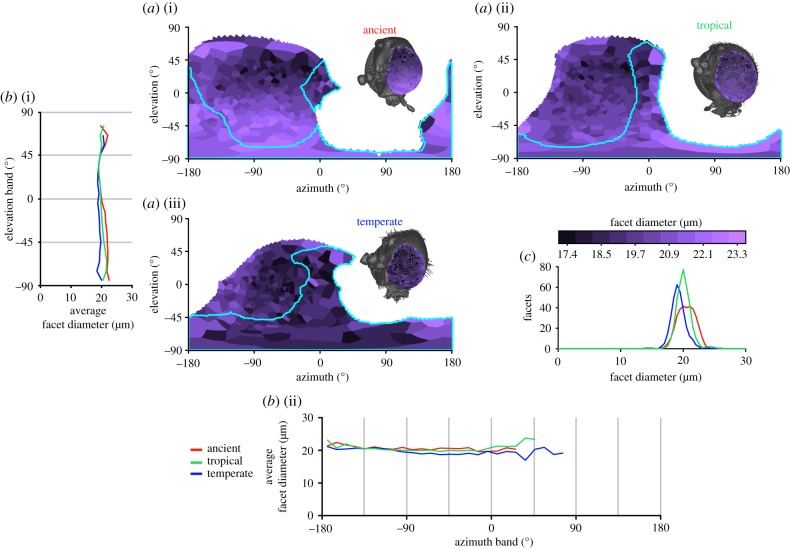
Facet diameter comparison between fungus gnat species. (*a*(i)) Ancient gnat, (*a*(ii)) extant tropical gnat and (*a*(iii)) extant temperate gnat. Details as in figure 3. Larger facet diameters indicate greater optical sensitivity. (Online version in colour.)

In the specimens we analysed, the cornea was the only intact part of the eye, but this could nonetheless be used to determine facet diameter, the local IF angle, and the full CP. The normal vector (NV) of each facet (figure 1*a*(vi)) and its viewing direction relative to the head (figure 1*a*(vii)) were calculated from the surfaces after importing them into Matlab (Mathworks Inc., USA). The limits of the visual field of each eye were calculated from the NVs of their outermost facets. The complete visual field and binocular overlap were then determined by combining the limits of both eyes (figure 1*a*(vii)). Facet diameters were calculated by finding the average distances between the centre of each facet and its neighbours (figure 1*a*(viii)) and IF angles were calculated by finding the average angle between the NV of a facet and its neighbours (figure 1*a*(viii)) [26]. The diameter of the facets provides an indication of the amount of light that is focused onto the underlying photoreceptors (e.g. larger facets capture additional light in proportion to the square of their diameter and increase optical sensitivity considerably) and can thus be used to approximate optical sensitivity. The IF angle (calculated using the surface NV of each facet; figure 1*a*(viii)) can be used to approximate the visual resolution, because each lens focuses light from a specific region of space (its viewing direction) centred about its optical axis. The smaller the angle between the optical axes of adjacent facets, the finer the visual resolution is likely to be [26]. The CP for each insect was compared between gnats in a common set of world-centric coordinates and integrated over azimuth and elevation in 10° bands of visual space (figure 2). Additionally, diagrams of how facet diameter and IF angle varied over the CP were generated from the visual axes and properties were calculated for each gnat. A Voronoi diagram was drawn around the visual axes of each eye, and the cells were coloured according to the local or IF angle (figure 3*a*) or facet diameter (figure 4*a*), which could also be depicted by colouring the individual facets of the compound eye (figures 3*a* and 4*a*, insets), or averaged over azimuth and elevation (figures 3*b* and 4*b*). This is an adaptation of the analysis procedure we recently developed [17], with a key conceptual difference being that here calculations are performed on individually labelled facets, rather than being interpolated between sparse labels.

**Figure 5.**
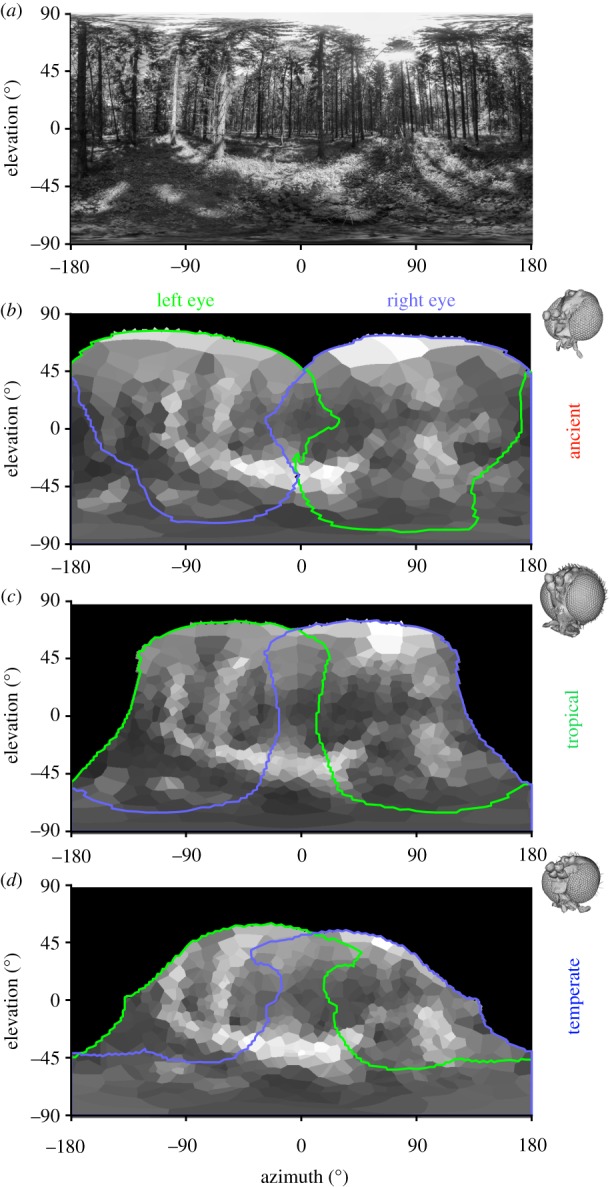
Simulation of how a forest scene could have been resolved through the eyes of different gnat species. (*a*) An equirectangular projection of a 360° panoramic image of a European forest in summer (image: istock.com/Bestgreenscreen; converted to greyscale). Simulated view of the scene with data quantified from the eyes of the ancient gnat (*b*), the extant tropical gnat (*c*) and the temperate gnat (*d*). The green and purple lines denote the limits of the corneal projection (CP; approximating the full field of view) for the left and right eyes, respectively. The simulation accounted for the variation in IF angle across each eye's CP, but does not account for optical, contrast or colour sensitivity. (Online version in colour.)

A simulation of how each gnat may have viewed a forest scene was generated from a panoramic forest image, considered to represent the type of scene that these insects may have viewed during their lifetime. In the simulation, we assumed that each facet accepted light over an angle equal to its IF angle, and then coloured the Voronoi cells based on the weighted average of the intensity value of the pixels that lay within this angular range (figure 5). For additional information on these steps, see the ‘Detailed computational analysis procedure’ section in the electronic supplementary material.

## 3. Results

Using our analysis technique based on microCT images of the heads and eyes of insects, we calculated the visual properties of fungus gnat eyes from the three-dimensional structure, shape and size of each individual corneal facet (figure 1*a*). To explore whether the visual systems of fungus gnats have changed across both habitat type and time, we applied our technique to three species (figure 1*b* and table 1). The exterior corneal surfaces reconstructed from microCT showed that, superficially, the ancient gnat appears most similar to the extant temperate species (figure 1*b*). While its head and eye size lie between those of the two extant gnats (table 1), the ancient gnat has a substantially larger monocular and total CP (indicating a larger field of view) than either extant species (figure 2*a* and table 2). The monocular CPs of both tropical gnats also extend further dorsally—to greater than 65° elevation (el.)—than the temperate species (figure 2*a*(i)), while the ancient gnat's monocular CP also extends posteriorly to beyond −180° azimuth (az.; figure 2*a*(ii)). Interestingly, the CPs of all species have a binocular overlap directed ventrally and frontally (figure 2*b*)—as elevation increases, these regions appear distinct as the binocular CP narrows at the horizon (approx. 0° el.) or separates completely in the case of the ancient gnat (figure 2*b*(iii)). The extant temperate gnat has the largest binocular overlap despite having the smallest total visual field (table 2), and its monocular and binocular CPs cover a larger portion of the ventral visual field than either tropical species (less than −45° el., figure 2*a*(i), *b*(i)).

**Table 2.** Summary of visual parameters of each fungus gnat.

species	*N. modesta* (temperate)	orfeliine species (ancient)	*Rutylapa* sp. (tropical)
left eye facet no.	303	333	367
right eye facet no.	296	335	373
left eye CP (%)^a^	48	55	46
total CP (%)^a^	69	96	78
binocular CP (%)^a^	27	17	15
facet diameter, *D* (μm)^b^	19.2 ± 1.0 (14.7–23.4)	20.5 ± 1.3 (16.8–25.6)	20.1 ± 1.0 (17.7–25.1)
IF angle, *ΔΦ* (°)^b^	10.5 ± 4.5 (3.9–40.1)	11.3 ± 3.7 (6.0–28.5)	9.1 ± 2.8 (4.3–20.6)
eye parameter, *P* (μm . rad)^b^	3.6 ± 1.6 (1.3–14.1)	4.1 ± 1.4 (1.9–12.5)	3.2 ± 1.1 (1.4–9.0)

^a^Corneal projection (CP) is indicated as a percentage of the visual sphere viewed.

^b^The mean ± 1 s.d. is provided for the facet diameter, inter-facet (IF) angle and eye parameter, followed by the measured minima and maxima values in parentheses.

Despite the gnats’ small eyes and limited number of facets, it is evident that they possess regional visual specializations. For example, the average IF angle varies across the elevation and azimuth profiles of all gnats (figure 3*b*). The lowest IF angles are directed mediolaterally, centred at approximately 0° el. and −75° az., which is approximately the centre of their monocular CPs (figures 2*a* and 3*a*). Although the largest IF angles occur at the periphery of each eye's CP (figure 3*a*), a surprisingly large range of IF angles extend to the periphery of each eye (figure 3*c*): the average elevation or azimuth IF angles at the periphery (15° or greater; figure 3*b*) are 50–100% higher than the mediolateral average (10° or less). Indeed, the smallest and largest IF angles of the temperate gnat differ by a factor of nearly eight (a 36.2° difference; table 2).

Although the facet diameters of each gnat's eye are relatively similar (figure 4*c* and table 2), a weak ventral-to-dorsal gradient of the average facet diameter is also visible in the elevation profile of both tropical gnat species (figure 4*b*(i)), with the average facet diameter dropping from 22 µm at −65° el. to 19 µm at 35° el. (above which facet diameter increases slightly). By contrast, the temperate gnat's average facet diameter remained relatively consistent over its elevation profile but appeared slightly higher along the posterior border of its visual field (less than −90° el., figure 4*a*(iii)). While regional variation of the facet diameter is present in the gnat eyes, this appears relatively minor compared with the variation observed in IF angle.

To better understand the consequences of the variation in corneal facet morphology that we observe across the fungus gnat eyes, we also conducted a simulation of the spatial sampling of each gnat species (figure 5). While this simulation provides only an approximate representation of how the different fungus gnats may have perceived their world, it is clear that all three species could obtain coarse, but nonetheless distinct, spatial information from the features in a forest scene. For instance, sunlight patches of forest floor and trees are relatively distinct, while the darker silhouettes of trees are also apparent. Although a clear horizon is not apparent, a vertical brightness gradient is present, indicating that the gnats would have had sufficient information to orient themselves appropriately with respect to the ground.

## 4. Discussion

In this study, we investigate the visual world of fungus gnat species from different locations and evolutionary periods to explore if these diminutive dipterans possess visual specializations for guiding behaviour in their challenging forest habitats. This required non-destructive analyses of rare and fragile specimens in different states of preservation, which we were able to do using a method based on microCT data. We show that our approach can be effectively applied to the cornea of fresh, dried or even fossilized compound eyes and be used to make calculations from individual facets (made feasible by the relatively low number of facets in the fungus gnat eyes), rather than being interpolated between sparsely distributed points across the eye. Our analyses show that fungus gnats do indeed have variations across their eyes and provide preliminary evidence that these variations represent visual specializations that may also vary between species from different geographical locations and across evolutionary time.

It is important to note that our data provide only approximations of visual parameters because we are limited to taking measurements from the cornea, whereas other optical elements below the cornea also play an important role in the properties of vision across the eye. For example, sensitivity is also affected by the receptor dimensions and the optical acceptance angle (variables that cannot usually be measured in natural preserved specimens because of degradation of internal structures) [26]. Discrepancies between the IF and the inter-ommatidial angle can also arise if the cone and rhabdom that underlie the lens are skewed relative to its NV [19]. It must also be noted that the variable preservation states of samples in historic collections may influence the results of this technique, and it is preferable to only analyse samples where the cornea is preserved entirely and not deformed. In this study, we superficially examined several samples from each collection to select those that appeared distortion free and believe that samples we imaged faithfully represent the typical eye shapes observed for each species. However, subtle cuticle distortions could occur in a way that differs between preservation techniques and we cannot exclude the possibility that such differences may have influenced our results. In this study, one of the main aims was to demonstrate the adaptability of our method when applied to different preservation techniques and this in part limited the choice of specimens analysed. Future studies may consider selecting samples preserved using a single technique, or explicitly comparing individuals of the same species preserved using different techniques.

While not readily apparent from their external morphology (figure 1*b*), we find visual specializations across the eyes of fungus gnat species from different locations, habitats and historical time periods. In particular, we identified two visual specializations common to all three of the Orfeliini tribe species investigated. The first is that their smallest IF angles are directed laterally (greater than 45° az.) and lie outside of their binocular field of view (figure 3*a*). This is in contrast to the larger Diptera that have been studied to date (including *Drosophila*), where the highest resolution is directed frontally [27–29]. It is not clear why the highest resolution regions of the fungus gnat visual field would be directed laterally. Possible functions may be related to improved horizon detection in the forest habitat, where a clear visual horizon might be difficult to detect because of occlusion by trees, or to better detect image motion created by nearby obstacles in order to avoid collisions and control flight.

Unexpectedly, all fungus gnat species investigated here have a large binocular overlap that is primarily directed ventrally and somewhat frontally (figure 2*b*). Although binocular overlaps have been measured in larger (female) flying insect species using our method [17] and the pseudopupil technique [20,30,31], these regions are typically smaller and approximately ventrally-to-dorsally symmetric. While ventral binocularity has not, to our knowledge, been considered previously, we propose that it may function to enable insects, particularly those with small eyes and therefore highly constrained sensitivity, to detect features on the ground in dim habitats. Information obtained in the regions of ventral binocular overlap could be integrated between both eyes, which would help to improve the signal-to-noise ratio of visual information on the forest floor.

Our results also indicate that the fungus gnat species studied here have distinct *differences* between their visual morphologies. We find that both the extant and ancient tropical gnat species have larger, more dorsally (and, to some extent, posteriorly) directed CPs than the temperate species (figure 2*a*). Despite having a smaller CP, however, the temperate gnat had a larger binocular overlap (27% versus 17% and 15% of the visual sphere for the temperate, ancient and tropical gnats, respectively; table 2) that is primarily directed ventrally (figure 2*b*). With respect to facet diameter, the tropical gnats had facets that were marginally larger than those of the temperate species (figure 4*c*) although their vertical gradient of facet size (not pronounced in the temperate species) results in a 3 µm reduction (approx. 2.5 s.d.) in facet diameter between their ventral and frontal visual fields (across 100° in el.; figure 4*a*(i)). These larger ventral facets may represent a regional visual specialization in the eyes of tropical gnats that would likely improve the optical sensitivity of the ommatidia viewing the forest floor. An analogous, albeit larger, increase in eye ‘regionalization’ is also found in the facet diameters of extant damselfly species that live in dark or visually complex habitats [3].

Our original hypothesis was that the eyes of fungus gnats from tropical forests would show specializations focused on improving optical sensitivity, and that these would be directed dorsally. Our predictions appear to be partially supported: both tropical species displayed a vertical gradient in facet size that would increase their sensitivity in the *ventral* (rather than dorsal) visual field. The increased sensitivity provided by the combination of both larger ventral facets and binocular overlap (the latter being common to all species) could enhance the reliability with which tropical gnats can discriminate objects on the dark rainforest floor. Less distinct but nonetheless detectable regional specializations can also be seen in the dorsal visual field—the position of both tropical species' eyes would enable them to capture light from a greater fraction of the dorsal world (at the expense of reducing the region of binocular overlap), which may facilitate sky-compass-based navigation under dense canopy cover [32]. The indication of increased optical sensitivity in the ventral visual field of the tropical fungus gnats in comparison with their temperate relative is consistent with our predictions and may thus be an indication of habitat-specific specialization, although more detailed studies are necessary to confirm if this is the case and if other visual adaptations occur between these environments.

Owing to the lack of a well-resolved phylogeny for the Orfeliini tribe [4], it is not possible to determine how much the common visual specializations that we observe in our study species are related to phylogenetic constraints and how much they are related to similarities in ecology, or to our limited sampling. The facets of cave-dwelling adult glow worms (*Arachnocampa luminosa*, from an adjacent sub-family of Keroplatidae) have larger heads (width: approx. 650 µm, versus approx. 450 to approx. 550 µm in our gnat specimens) and eyes than our gnat specimens. The facets of *A. luminosa* are also proportionately larger (diameter: 27–28 µm, versus approx. 19 to approx. 21 µm) but disproportionately more numerous (approx. 750, versus 296 to 373) [33] than in the fungus gnats. Although glow worms are phylogenetically close to Orfeliini, they inhabit dim caves—an environment that is unusual for dipterans. The differences between the glow worm eyes and the forest-dwelling fungus gnats are consistent with the variation expected if the vision in these species was being shaped by their visual environment, supporting our hypothesis that habitat may indeed shape the visual specializations of this dipteran family. We are not aware of any studies on the eyes of other species in the superfamily Sciaroidea against which we can further explore the phylogenetic influence of visual specializations in fungus gnats, although behavioural experiments also indicate that dark-winged fungus gnats (*Bradysia* sp., in the family Sciaridae) have high optical sensitivity, as they display phototactic behaviour at light intensities less than 5 lux [34]. Given the high energetic cost of vision [35], the specializations observed in our Orfeliini tribe species are likely to also represent functional adaptations and will, at the very least, influence how the gnats perceive their world.

The ability of our technique to simulate vision through an arthropod's eyes provides insights into how it views a particular scene. Given the substantial differences between arthropod vision and our own, we believe that this is a valuable tool for interpreting visual parameters and for developing hypotheses about the function of specific visual adaptations (figure 5). For instance, despite being weak fliers, fungus gnats are effective pollinators [36]. Understanding how gnats view their world might help us to understand how small insects detect flowers in a variety of forest habitats and, in turn, understand how habitat changes might affect this ability. Gaining knowledge about the visual capabilities of gnats and other insects and being able to simulate their visual world is a valuable starting point for designing behavioural assays to test visual capabilities of species that inhabit different habitats. This, in turn, may provide important information for the development of effective species-conservation strategies, which are sorely needed considering the current drastic declines of flying insect biomass due to habitat change [37].

Well-preserved amber endocasts of insects from the Early Cretaceous [38] are available in museum collections and provide a rich resource of samples which, with the help of our technique, can now be used to study the evolution of specific visual traits across a broad range of taxa. For example, prominent acute zones with regions of flattened and enlarged facets have evolved to accompany pursuit behaviour in many insect species. Identification of such regional specializations in the fossil record could, for instance, be used to investigate the evolutionary origins of pursuit mating strategies in male bees [39]. Fine retinal structures can be identified in our ethanol-preserved sample (electronic supplementary material, figure S2) and are also likely to be visible in images of exceptionally preserved amber inclusions [40]. MicroCT is often used to describe the morphology of fossil insects for taxonomic purposes (e.g. [41,42]) and data obtained for systematics could also be used to describe a specimen's vision. Applying our methodology to the substantial range of dried specimens collected over the last several centuries would also facilitate investigations into the influence of recent anthropogenic disruptions on the evolution of invertebrate visual systems. For example, it could be used to examine whether the increase in light pollution over the last century has placed any selection pressure for specific visual traits on the eyes of nocturnal urban insects [43,44]. Testing these and many other hypotheses related to the factors driving visual evolution is now possible by applying our technique to analyse the compound eyes of the arthropod specimens available in museum collections.

Overall, the results of this study demonstrate that our analysis method can be used to identify visual specializations present in species from different times and environments and in different states of preservation. We acknowledge that our focus on rare specimens naturally limits the scope of this current work, and also that the limited (but unavoidable) sampling of this case study provides only tentative support for our hypotheses. Yet, these preliminary results provide inspiration and justification for a larger, more detailed study considering variation both within species and within habitat groups while controlling for phylogenetic effects. A distinct advantage of our method is that it facilitates direct comparisons of vision between species. Unlike existing approaches, the method enables the visual properties of each insect to be presented in a common, world-based coordinate frame, even if there are substantial differences between the visual morphology and size of the eyes [17]. This means that it is possible not only to make comparisons across differently sized individuals from the same species and between individuals from different species, but also to make comparisons across taxa. We therefore hope that this study will provide inspiration for future investigations into the visual systems of rare or extinct insect taxa.

## Supplementary Material

Supplementary methods

## Supplementary Material

Supplementary figures

## Supplementary Material

Supplementary tables

## Data accessibility

Data available from the Dryad Digital Repository: https://dx.doi.org/10.5061/dryad.s502854 [45].
