# Supplementary material for: Exploring the visual world of fossilized and modern fungus gnat eyes (Diptera: Keroplatidae) with X-ray microtomography
Source: J R Soc Interface. 2020 Feb 5;17(163):20190750. doi: 10.1098/rsif.2019.0750 (PMC7061697; doi:10.1098/rsif.2019.0750)
Supplement: Supplementary methods [file rsif20190750supp1.docx]

Supplementary Methods

*Detailed surface analysis procedure*

Each image stack from microCT was imported into Amira (v6.2, FEI), within which an interactive workflow was developed that allowed us to create a surface defining the head of the scanned insect, and then to delineate the border of the left eye plus each of its facets. After labelling the facets, these data were mirrored to the right eye, and the surface data and mirror transform were then exported to Matlab (Mathworks Inc.) for further computational analysis (see the following section). In the following descriptions, windows within the Amira console are indicated in *italics*, Editor windows (accessible from the *Properties* *View* of a module) are indicated by underlining, and further modules, tools, and options are indicated in ‘single quotes’.

The head surface was created by first labelling the voxels within the head volume, and then fitting a surface around their border, as described in the following. The ‘Threshold’ tool in the *Segmentation View* was used to select and label the voxels that were part of a gnat (which were lower intensity than the surrounding material for the amber-embedded specimen, but higher in the other two cases). The ‘3D Lasso’ tool was then used to select and remove excess labelled material, such that only the fungus gnat’s head capsule remained labelled. Following this, a surface was created around the labels using the ‘Generate Surface’ (a ‘smoothing extent’ of 2 was used when generating the surface) and ‘View Surface’ modules in the *Project View*. Sometimes the surface created for the eyes appeared to be poorly formed, particularly for the amber-embedded and alcohol-preserved samples. In the former case, this was because decayed material from the gnat coated the inside of the endocast, and often had higher absorption than the amber. In the alcohol-preserved case, it was because of relatively low absorption contrast between the cuticle and the alcohol. In both cases, the ‘Brush’ tool was used to manually correct the label field of 2D slices in the *Segmentation Editor*, to ensure that individual facet surfaces of the left eye appeared smooth in the generated surface and, additionally, that the surface of the eye was closed, with no gaps present between facets. The scan of the dried gnat had high contrast between the cuticle and air and the initial surface that was generated did not require such corrections.

After completing any required corrections, a final surface was created and a ‘Create Surface Geodesic Path’ module was attached to it (all remaining steps are performed in the *Project View*). In the Surface Path Editor, the connector was set to ‘dijkstra’, and the control points to ‘vertex’. The Surface Path Editor allows points to be selected around the border of the left compound eye, which were linked by paths taking the shortest route across the surface between sequential points. To isolate this area from the remainder of the surface, after closing the path around the eye, the ‘Patchify Surface’ was selected. The Surface Editor was then used to selectively display only the compound eye patch, after which the ‘Extract Surface’ module was used to create a new surface only including the compound eye. A second ‘Create Surface Geodesic Path’ module was then attached to the isolated corneal surface and paths were traced in a circle around the base of every facet on the compound eye. Approximately 60 control points were placed per facet and closing a path upon completion allowed a new path to be started for the next facet. Note that each fungus gnat facet has a dome shaped cornea; many other insects have flatter, hexagonal lenses and placing a control point at each of the six corner points of a facet would probably be sufficient in such cases. The right eyes of gnats were not individually segmented, but we counted the number of facets by using another geodesic path to place control points at the centre of each facet. This indicated that the gnat eyes were approximately symmetrical, with at most a 2.4% difference in facet number between the eyes (table S1).

Each head surface was then aligned using the Transform Editor, by positioning and aligning it such that it faced forwards in the default XY image in the *Project view*. The head was aligned in its frontal (yaw) and axial (roll) planes based on its symmetry. The pitch (sagittal plane) of the head could not be aligned by symmetry, but was positioned such that each head appeared to have a similar, forwards facing, orientation (figure 1*b*) [1]. While the orientation of the head may vary during flight this provides a consistent reference frame between insects. After aligning the head, the resulting rigid transformation matrix was copied to both the isolated eye surface and the paths around each facet. While the right eye was not separately labelled, we determined the transformation required to mirror the labelled left eye such that it was aligned with the position of the right eye. To mirror the left eye, we used the ‘Scan surface to volume’ module on the left eye surface to create a volume in which the voxels corresponding to the surface were labelled. The ‘Resample transformed image’ module was then applied to this volume, which created a second volume, the axes of which were orthogonal to the world coordinate frame. This volume could then be mirrored around the sagittal plane of the head by using the ‘Flip X’ button in the Crop Editor, and a ‘Volume rendering’ module was used to visualize the position of the mirrored eye. Finally, the Transform Editor was used to manually adjust the alignment of the mirrored right eye such that it visually matched the position of the actual right eye on the surface of the head as closely as possible.

After completing the above-described processing workflow in Amira, it was necessary to export data for use in Matlab. Both the head surface and eye surface were exported as ‘stl’ files in little endian format. The paths surrounding each facet were exported as ‘Amira lineset’ files in asci format. To facilitate importing paths into Matlab, it was necessary to split the lineset file into two parts using a text editing program. Each file contains data under two headings; @1 is a list of indices indicating the vertices to use for each control point in an individual path (-1 indicates that start of a new path), @2 is a list of 3D vertices coordinates referenced by the indices. It is necessary to copy the indices and vertices into individual text files. Finally, the 4x4 transform matrices for the head and the right eye were recorded by using the ‘GetTransform’ command in the *TCL Console*, and the ‘Max index’ and the ‘Min coord’ values of the right eye volume were recorded from Crop Editor.

An example of the above Amira analysis procedure applied to *N. modesta* is provided in the ‘Amira demo’ folder at the Data link below. The data exported from Amira for import into Matlab are also contained in separate folders at the Data Link.

Data Link: <https://doi.org/10.5061/dryad.s502854>

*Detail computational analysis procedure*

The data exported by Amira was imported into Matlab and analysed using a series of custom written scripts to quantify the visual properties of gnat eyes and perform a visual simulation. The analysis procedure involves applying the rigid transformations to the imported surfaces and identifying the facet surfaces from the path points. The corneal normal vector (NV) from each facet was calculated, which was used to quantify the eye’s visual parameters and fields of view [1]. Various plotting options were implemented for the data, including performing a visual simulation of the insect’s perspective of a scene. This analysis was mostly automated, and here we describe the analysis steps performed by the script. Variable names that the user may wish to modify are indicated in ‘parenthesis’, and influence data importing and various display options as outlined in the initial ‘Configuration’ section of the supplementary ‘AnalyseCorneaMain.m’ file.

Data were initially imported into Matlab based on the user specified files in the initial portion of the ‘Configuration’ section. The exported transformations were applied to the surfaces such that they were represented in the same orientation as seen in Amira. The paths were initially formatted as a single list of points, but we separated this into an individual list of vertices for each facet’s border. After this, the coordinates on the eye surface enclosed by each border path were selected as the corneal surface for that facet. The centre point of each facet was selected as the surface point that lay closest to average coordinate of the border path (in a 2D projection), and then the NV of the cornea was calculated from this point. This calculation involved fitting a second order polynomial surface to the corneal surface vertices, and calculating the normal direction from the derivative at the central point [1, 2]. The NV was taken to indicate the optical axis of the facet. The same procedure was performed for the right eye using the facets mirrored from the left eye.

The neighbours of each facet were determined based on the shortest distances between its centre and those of each other facet (each facet linked to six neighbours, unless it was on the border of the eye). Having established the neighbours of each facet, the inter-facet (IF) angle of a given facet was calculated as the average angle between the normal from its centre and those of its neighbours and the facet diameter was calculated as the average distance from its centre point to those of its neighbours. The eye parameter, was calculated by multiplying each facet’s IF angle by its diameter [3]. Note that the calculating the eye parameter assumed that the inter-ommatidial angle equals the computed IF angle, which may not be true and limits the accuracy with which we can report this parameter. It is possible to perform a local smoothing operation on the data, whereby the NVs and diameters of each facet are averaged with those of their neighbours. Smoothing may be useful if the visual parameters of an eye vary irregularly and can be adjusted using the ‘smoothingFactor’ variable (we did not smooth the results shown in this study).

To approximate the field of view of each eye, we determined its corneal projection (CP). We did this by first calculating the intersection of each NV on a distant sphere. We then discretized the sphere into many equally-spaced points and determined which of those points lay within a boundary enclosing the NV intersections. The points within the CP were determined in this manner for each eye individually. The union of these two sets of points provided the total CP, while the intersection indicated the portion of the CP with binocular overlap. The borders around each region were also calculated for use in the later plotting steps; note that if multiple individual regions are present within a given CP (such as in the binocular CP, figure 2*b*), the border calculation may take a long time to compute. The visual sphere was also divided into individual regions for each facet. This was calculated either for the facets of the left eye only (figures 3 & 4) or for both eyes (figure 5) by calculating the borders of the voronoi cells (on the surface of the sphere) around the sphere intersection points of the facets. Although the voronoi cells covered the entire sphere, those on the borders were limited to the extent of the previously calculated CP.

Different methods were implemented to plot the data resulting from the previous analysis. The individual NVs and CPs were drawn onto a sphere surrounding a visualization of the head surface (figure S2*a*). The values of each calculated parameter were encoded using colour maps, and displayed either as a world-based representation by colouring the voronoi cells of the left eye (figures 3*a* & 4*a*), or as a head-based representation by colouring the corneal facets on the eye surface (figures 3*a* & 4*a*, insets). While the voronoi cells were calculated on a sphere, we displayed them as 2D maps using an equirectangular projection, onto which either the binocular CP or the right eye CP could be displayed using the ‘plotBinoLine’ and the ‘plotRightEyeLine’ variables, respectively. It was also possible to draw contour lines of one parameter upon the map of another using the ‘dispContourOnInterOImage’ variable (for example contours of facet diameter on a map of IF angle, figure S2*b*). Facets viewing the binocular CP could also be indicated with markers on the head centric representation (using the ‘binoMarkerSize’ variable). All plots could be saved automatically by setting the ‘saveImages’ flag.

To display colour maps, the observed range of each calculated parameter was discretized into a number of equally spaced bins (set with ‘numColBins’ variable). By default, this discretization is performed for each insect, and different insect eyes had different ranges and receive different colour mappings. To compare between multiple insects, ‘AnalyseCorneaMain.m’ should first be run for each insect with the ‘saveData’ flag set. This will save the calculated parameters for each insect analysed and the ‘PlotGroupHistograms.m’ script (which requires similar variables to be set as in the main analysis file) was used to produce overlaid histograms for the parameters, and to compute the mean, standard deviation, and range of each parameter for each insect. The latter file also determined the total ranges of each calculated parameter between all data sets and used them to compute the appropriate discretization for all insects. In the original file, the ‘useGroupColBins’ flag should then be set, and re-running the main analysis file for each insect creates plots with a common colour mappings.

A simulation of an insect’s vision of a user-supplied panoramic image (figure 5) or a chequered sphere (figure S2*d*) could also be computed (choose by setting the ‘simUserIm’ flag). When using the chequered sphere, the number of chequers was set using the ‘numChequers’ variable (note that a higher number of chequers results in a lower wavelength and higher spatial frequency) and that chequers are mapped onto equirectangular image and do not represent equal angular areas on the world sphere (figure S2*c*). The simulation was performed using a direct RGB-to-grayscale conversion from the image provided as no knowledge of the spectral sensitivity of the insects was available. The world coordinates were determined for each pixel of the image being used, and we simulated the insect’s position at the centre of the world sphere. For each facet, we averaged the intensity of all points that lay less than one IF angle from its corneal axis and weighted this average by a Gaussian kernel with full width at half maximum (FWHM) equal to the IF angle of each facet. This assumes that the acceptance angle equals the IF angle for each facet (this proportion can be adjusted using the ‘deltaPScale’ variable). We then shaded the facet’s voronoi cell with the averaged grayscale value, and the simulation could either be performed for the left eye only or for both eyes by setting the ‘simBothEyes’ variable. The shaded cells were plotted on an equirectangular image in a similar manner to the colour maps described above, and the border of the fields of view could also be displayed.

The Matlab scripts developed for this analysis are available from the Code Link below. Additionally, the ‘per facet’ calculation results are downloadable at the Data Link above.

Code Link: <https://github.com/gavinscode/museum-sample-eye-analysis.git>

**Supplemental References**

[1] Taylor, G.J., Tichit, P., Schmidt, M.D., Bodey, A.J., Rau, C. & Baird, E. 2019 Bumblebee visual allometry results in locally improved resolution and globally improved sensitivity. *eLife* **8**, e40613.

[2] Taylor, G.J., Ribi, W., Bech, M., Bodey, A.J., Rau, C., Steuwer, A., Warrant, E.J. & Baird, E. 2016 The dual function of orchid bee ocelli as revealed by X-ray microtomography. *Curr. Biol.* **26**, 1319-1324.

[3] Snyder, A.W. 1979 Physics of vision in compound eyes. In *Comparative physiology and evolution of vision in invertebrates A: Invertebrate photoreceptors* (pp. 225-313, Springer Berlin Heidelberg.
