# Supplementary material for: Exploring the visual world of fossilized and modern fungus gnat eyes (Diptera: Keroplatidae) with X-ray microtomography
Source: J R Soc Interface. 2020 Feb 5;17(163):20190750. doi: 10.1098/rsif.2019.0750 (PMC7061697; doi:10.1098/rsif.2019.0750)
Supplement: Supplementary figures [file rsif20190750supp2.docx]

Supplementary Figures


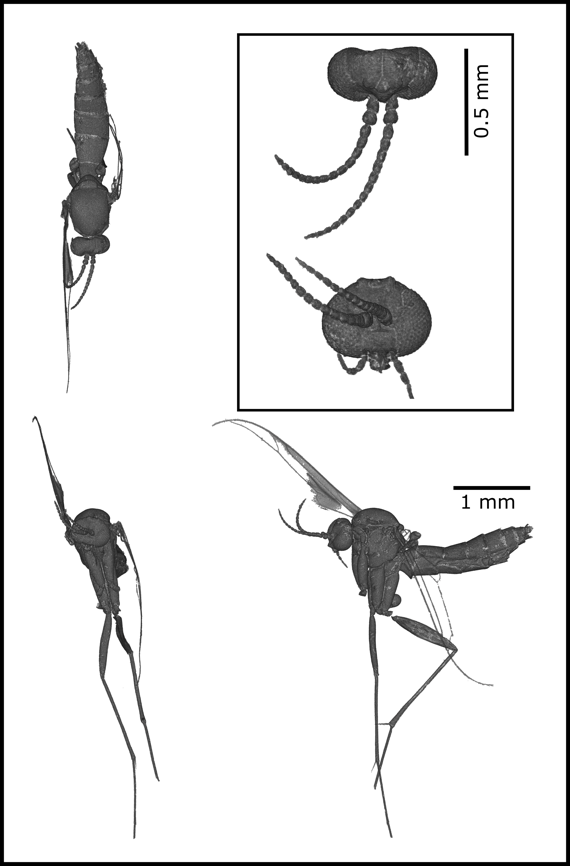


**Figure S1**: Habitus image created from the endocast of the female Eocene gnat specimen. Unfortunately, the degradation of the wings limited a full species identification of the sample, but the general morphological features place it in the tribe Orfeliini of the family Keroplatidae in the order Diptera.


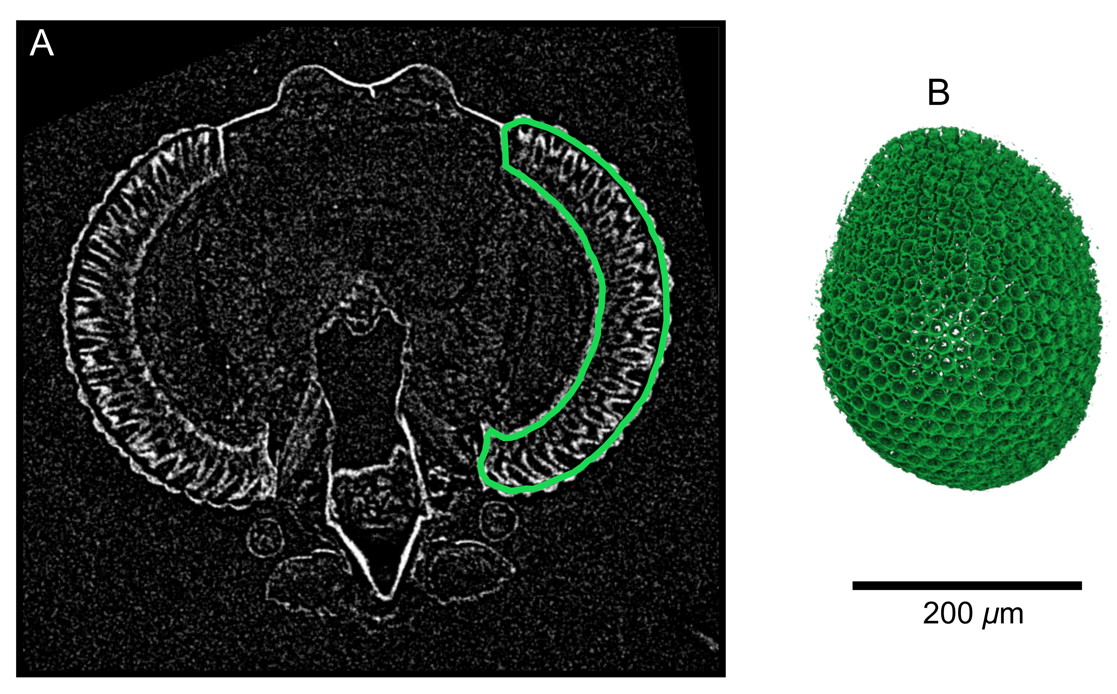


**Figure S2**: Internal structure of the alcohol-preserved *Rutylapa* sp. specimen. (*a*) A slice through the image volume shows that, in addition to the exterior cuticle, details within the retina of the eyes have high X-ray absorption. (*b*) We segmented the retinal volume of the left eye (enclosed by the green line in A), and used volume rendering to visualize the 3D retina. A conical X-ray absorbing structure is visible for each ommatidia, which may represent the secondary pigment cells surrounding the photoreceptors. The scale bar applies to both *a* and *b*.


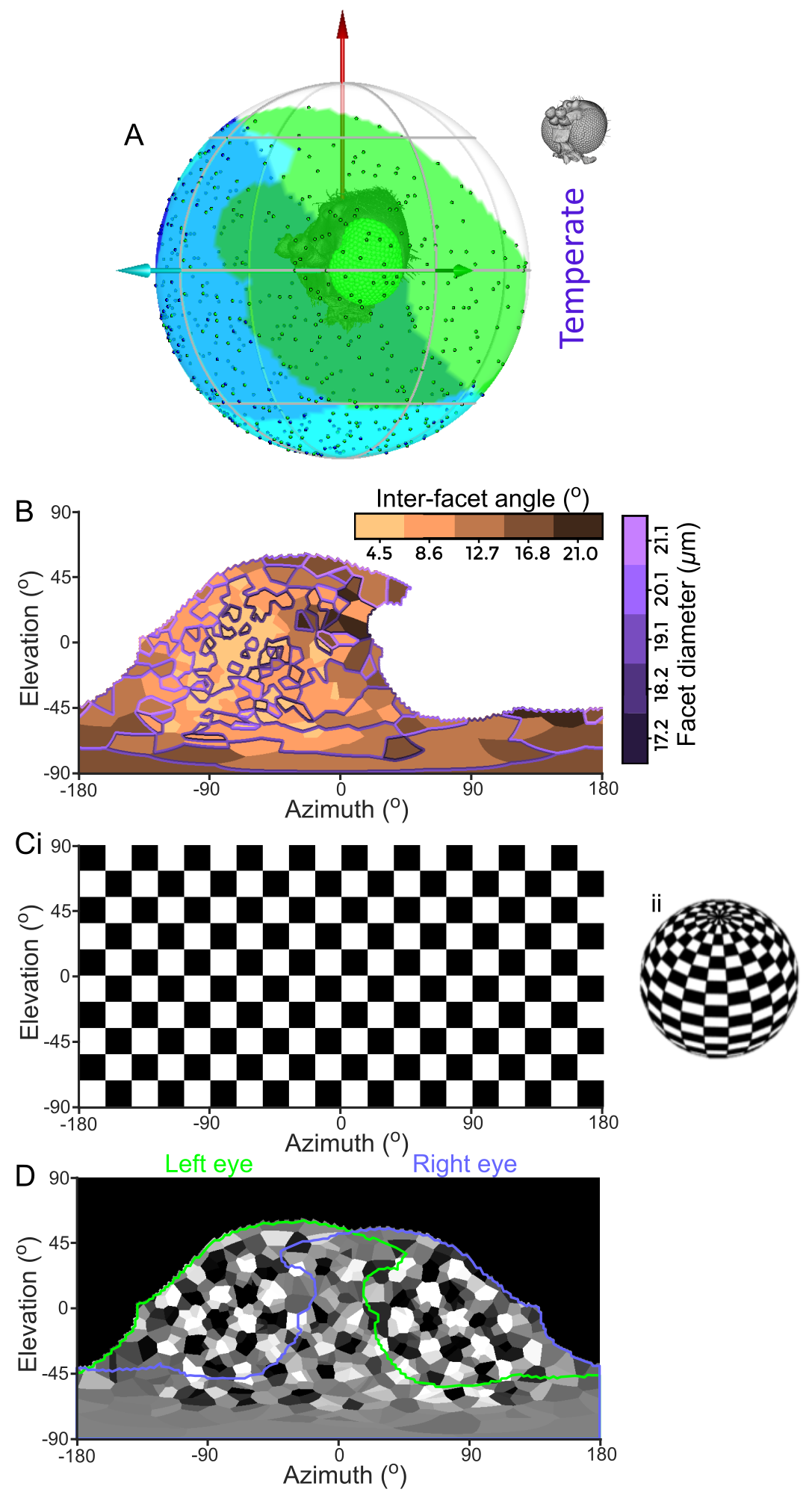


Figure S3: Examples of plotting options not used in the main text. (*a*) Head of a gnat plotted inside a sphere representing the world. Its left monocular, right monocular, and binocular CPs are represented by green, blue, and cyan shading on the sphere respectively. Points represent the intersection of the normal vectors (NVs) of individual facets on the world sphere (as in figure 1*avii*), and the green, cyan, and red arrows represent left lateral, frontal, and dorsal directions in the head-based reference frame. (*b*) Colour map of the inter-facet (IF) angle projection on the world, overlaid with coloured contours representing the projected facet diameters (note that the colour scaling used is different to that in Figs. 2 & 3). (*c*) Equirectangular projection (*i*) on a chequered sphere (*ii*) with 36° period. (*d*) visual simulation of the gnat vision as if it was located inside the chequered sphere. The green and mauve lines denote the limits of the corneal projection (CP) for the left and right eyes respectively. The data used in this figure was from the temperate species *Neoplatyura modesta*.
