# Supplementary material for: Exploring the visual world of fossilized and modern fungus gnat eyes (Diptera: Keroplatidae) with X-ray microtomography
Source: J R Soc Interface. 2020 Feb 5;17(163):20190750. doi: 10.1098/rsif.2019.0750 (PMC7061697; doi:10.1098/rsif.2019.0750)
Supplement: Supplementary tables [file rsif20190750supp3.docx]

Supplementary Tables

Table S1: The specific scan settings for the Zeiss XRM520 tomograph that were used for imaging. The 4x objective was used for all samples.

| Species | *Neoplatyura modesta* | Orfeliine species | *Rutylapa* sp. |
| --- | --- | --- | --- |
| Source voltage (kV) | 80 | 80 | 50 |
| Source power (W) | 7 | 7 | 4 |
| Exposure time (s) | 2 | 10 | 10 |
| Projections (#) | 1,601 | 2,001 | 1,601 |
| Source to rotation axis distance (mm) | 15.0 | 21.0 | 14.0 |
| Rotation axis to detector distance (mm) | 86.3 | 120.9 | 80.0 |

Table S2: Ascension numbers for micro-CT data used in this study

| Data | Images DOI | Labels DOI | Surface DOI |
| --- | --- | --- | --- |
| *N. modesta* - head | 10.17602/M2/M77216 | 10.17602/M2/M77217 | 10.17602/M2/M77218 |
| Orfeliine species – head | 10.17602/M2/M77219 | 10.17602/M2/M77220 | 10.17602/M2/M77221 |
| Orfeliine species – body | 10.17602/M2/M77342 | 10.17602/M2/M77343 | 10.17602/M2/M77345 |
| *Rutylapa* sp. - head | 10.17602/M2/M77222 | 10.17602/M2/M77223 | 10.17602/M2/M77224 |
